# Effect of triptorelin 3.75 mg subcutaneously injection every 6 weeks on adult height in girls with idiopathic central precocious puberty

**DOI:** 10.1186/1687-9856-2015-S1-P92

**Published:** 2015-04-28

**Authors:** Yan Liang, Hong Wei, Jie Li, Ling Hou, Jianling Zhang, Wei Wu, Yanqin Ying, Xiaoping Luo

**Affiliations:** 1 Department of pediatrics, Tongji hospital, Tongji medical college, Huazhong University of Science and Technology, Wuhan, Hubei Province, China; 2 Sichuan Provincial People's Hospital, Chengdu, Sichuan Province, China

## Aims

To evaluate the long-term efficacy of triptorelin 3.75 mg subcutaneously injection every 6 weeks on final height in girls with idiopathic central precocious puberty.

## Methods

Forty females with ICPP received triptorelin 3.75 mg every 6 weeks subcutaneously injection in our hospital from 2002 to December 2010 and reached final height were collected. These patients were divided into two groups according to whether there was presence of growth deceleration and rhGH used concomitantly during the treatment. Group A: triptorelin alone, n=17; group B: triptorelin + rhGH, n=23. During the treatment, height, weight, annual GV, sexual development, PAH and adverse effects were observed. BA and height SDS were monitored yearly. After discontinuation of treatment, follow-up was continued for 4~9 years till final height was attained, and age of menarche, time of menarche from discontinuation were recorded.

## Results

FAHs were 159.81±4.95 cm and 161.01±4.89 cm respectively in the two groups, exceed the genetic target height (THt), about the 50th percentile of normal female height. FAH increased by 1.51±4.30 cm, 4.86±4.49 cm from THt respectively. The values of (FAH-THt) showed significant difference between the two groups (p<0.05). FAH was less than the predicted adult height (PAH) before and after treatment in group A, and greater than that of group B. The value of (FAH-PAH post-treatment) showed significant difference between the two groups (p<0.05). FAH was positively correlated with Ht SDS-BA at the end of treatment, THt, course of rhGH treatment and age of menarche (r2=0.66). BMI increased after treatment compared with that before treatment in both groups, however, compared with healthy children at the same age, there was no significant tendency of increase. Ages of menarche were 11.74±0.66 years and 12.18±0.69 years respectively. Times of menarche from discontinuation were 17.41±6.96 months and 14.71±4.77 months respectively.

## Conclusion

The final adult height in patients with ICPP was improved effectively by triptorelin 3.75 mg subcutaneously injection every 6 weeks, and more height gain will be achieved when rhGH was used concomitantly to refrain from growth deceleration during the treatment. BMI maintained steadily and ovarian function restored quickly after discontinuation of the treatment with the age of menarche similar to that of normal children. Neither significant adverse effect nor polycystic ovary syndrome was observed.

